# Primary vaginal squamous cell carcinoma with bladder involvement in uterine prolapsed patient: Case report

**DOI:** 10.1097/MD.0000000000008993

**Published:** 2017-12-15

**Authors:** Tadeusz Fedus, Renata Raś, Mariusz Książek, Justyna Filipowska, Ewa Kaznowska, Andrzej Skręt, Joanna Skręt-Magierło, Edyta Barnaś

**Affiliations:** a Clinical Department of Urology and Urology Oncology; b Obstetrics and Gynecology Clinic, Frederick Chopin Clinical Provincial Hospital No 1; c Department of Patomorphology, Chair of Morphological Sciences, Faculty of Medicine; d Institute of Nursing and Health Science; e Institute of Obstetrics and Emergency Medicine, Medical Faculty, University of Rzeszow, Rzeszow, Poland.

**Keywords:** bladder involvement, uterine prolapsed, vaginal cancer

## Abstract

**Rationale:**

Primary vaginal squamous cell carcinoma (SCC) is a rare disease. Primary SCC in prolapsed vagina is extremely rare. In the presented case additional bladder involvement was found.

**Patients concerns:**

Primary vaginal SCC may be misinterpreted as decubitus in prolapsed vagina and it may delay proper diagnosis and treatment.

**Diagnoses:**

Diagnosis was confirmed by the vaginal ulceration biopsy and cystoscopic biopsy of the involved bladder.

**Interventions:**

In the case presented percutaneous nephrostomy was the only possible treatment of hydronephrosis.

**Outcomes:**

In advanced primary SCC (Figo IVA) with nodal involvement palliative treatment is only option.

**Lessons:**

Primary SCC mimicking decubitus which appeared in prolapsed vagina, may be accompanied by bladder involvement.

## 1 Introduction

Vaginal carcinoma is considered the rarest gynaecological neoplasm, accounting for only about 1% to 3% of all gynaecological malignancies.^[1,2]^ Vaginal carcinoma with prolapse is very rare, but in elderly women 13.6% to 16.3% of total vaginal cancer coexist with prolapse.^[3]^

Some authors report the metastases of vaginal cancer coexisting with prolapse to the chest, nodes, liver, iliac bone.^[3,4]^

To the best of authors’ knowledge, there is no case description with bladder involvement.

The aim of this report is to present the case of primary vaginal squamous cell carcinoma (SSC) with bladder involvement in uterine prolapse.

## 2 Case report

A 69-year-old patient (G6 P6) (height, 155 cm; weight, 66 kg) reported vaginal prolapse for several years and incontinence, abdominal pain and hematuria. Comorbidities: type 2 diabetes for 20 years, circulatory failure, paroxysmal atrial fibrillation, anaemia, urinary tract infection, hypertension, chronic biliary gastritis, cervical osteoarthritis.

**Figure 1:**
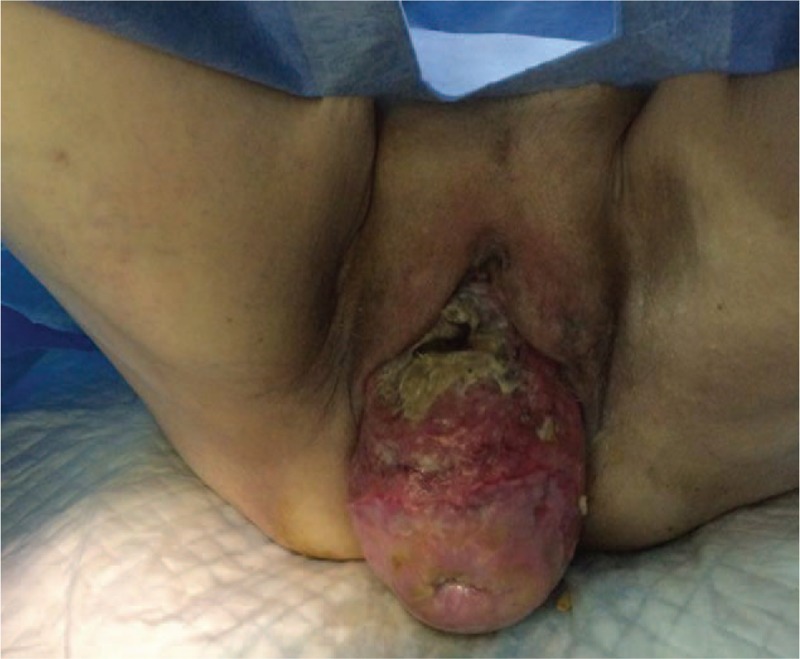
Irreducible vaginal prolapse with vaginal squamous cell carcinoma. Ulcurated lesion involving almost entire anterial vaginal wall.

**Figure 2:**
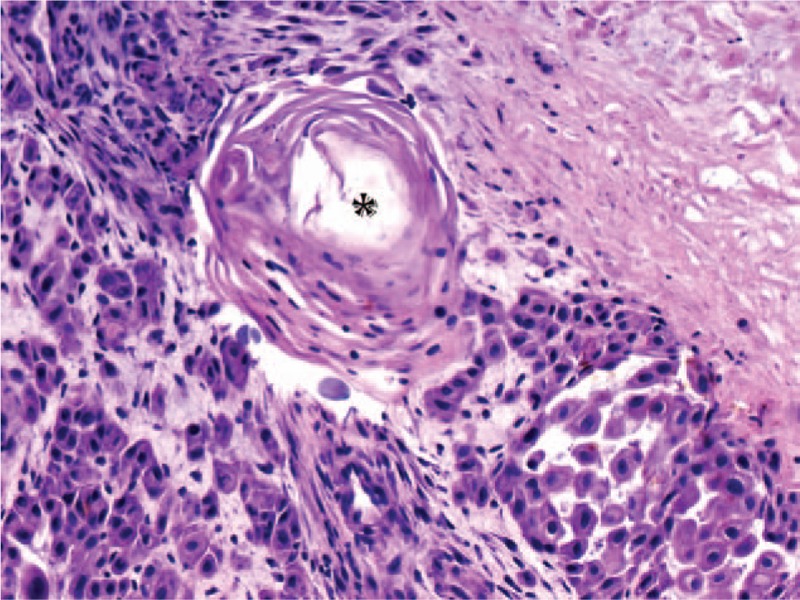
Surgical diagnostic biopsy of prolapsed vagina: well-differentiated keratinizing squamous cell carcinoma of vagina (H&E, objective ×20).^∗^ keratin pearl formation in SCC.

Physical examination on admission revealed anterior vaginal and uterine walls prolapse, with 8 cm in diameter large ulcerated lesion (Fig. 1) located on the anterior vaginal wall. Diagnostic biopsy of the lesion revealed squamous cell carcinoma (Fig. 2).

Additional tests: hemoglobin 11.0 g/dL (12.0–16.0); hematocrit 33.9% (37.0–47.0); erythrocytes 4.34 × 106/uL (4.00–5.00); MCV 78.1 fL (80–94); MCH 25.3 pg (27.0–31.0); MCHC 32.4 g/dL 931.0–37.0); RDW-SD 48.3 fL (36.4–46.3); RDW-CV 17.7% (11.7–14.4); Blood Plate 296 × 103/Ul (140–400); plate distribution width (PDW) 11.6 fL (9.80–16.20); MPV 9.8 fL (9.40–12.50); platelet large cell ratio (P-LCR) 24.1% (19.10–46.60); WBC 8.12 × 103/ul (4.0–10.0); Macro R 6.7%; Micro R 7.9%; total protein 7.1 g/dL (range 6.3–8.2); chlorides 112.0 mmol/L (98.0–107.0); activated partial thromboplastin time (APTT) 29.0 s (25.9–36.6); prothrombin time 9.2 s (7.6–11.4); Prothrombin index 103.7% (80.0–120.0); INR 1.0 (0.9–1.3); D-dimer 2 208.8 ng/mL (<500.0); fibrinogen 503.9 mg/dL (180.0–400.0); glomerular filtration rate (GFR) 27 mL/min/1.73 m^2^ (>60.0); glucose 115 mg/dL (70–99); creatinine 1.85 mg/dL (0.52–1.04); urea 66 mg/dL (15–43); potassium 4.9 mmol/L (3.5–5.1); sodium 144.0 mmol/L (137.0–145.0); TSH 0.50 uIU/mL (0.27–4.20).

**Figure 3:**
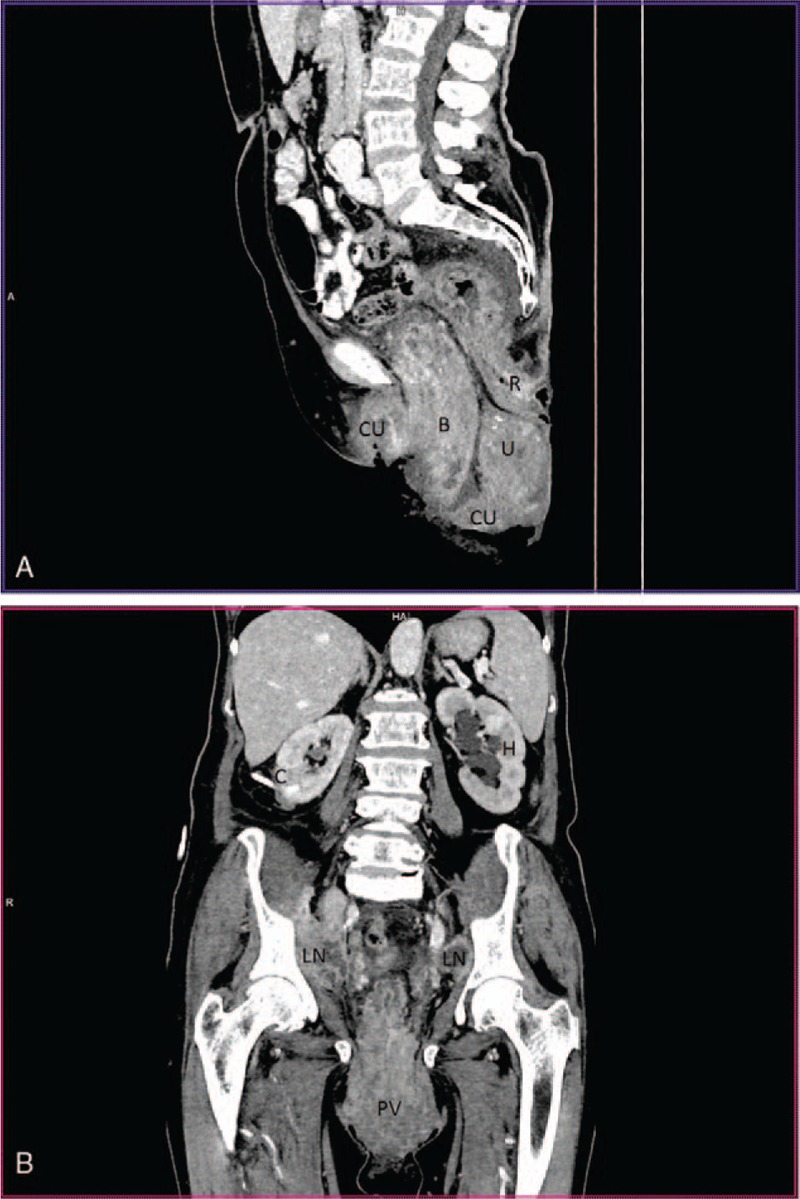
Sagittal pelvic computed tomography of the patient. In protruded vagina: bladder—B, rectum—R, carcinomatous ulceration—CU, uterus—U. Abdominal and pelvic frontal CT. PV—protruded vagina, LN—enlarged iliac nods, H—left kidney with hydronephrosis, C— nephrotomic catheter in right kidney.

Sagittal pelvic CT showed protruded vagina, infiltrated bladder wall, uterus, and rectum. In frontal CT scan of abdomen, enlarged pelvic iliac nodes, hydronephrosis of the left kidney with left hydroureter, in the right kidney nephrostomic catheter were observed (Fig. 3).

**Figure 4:**
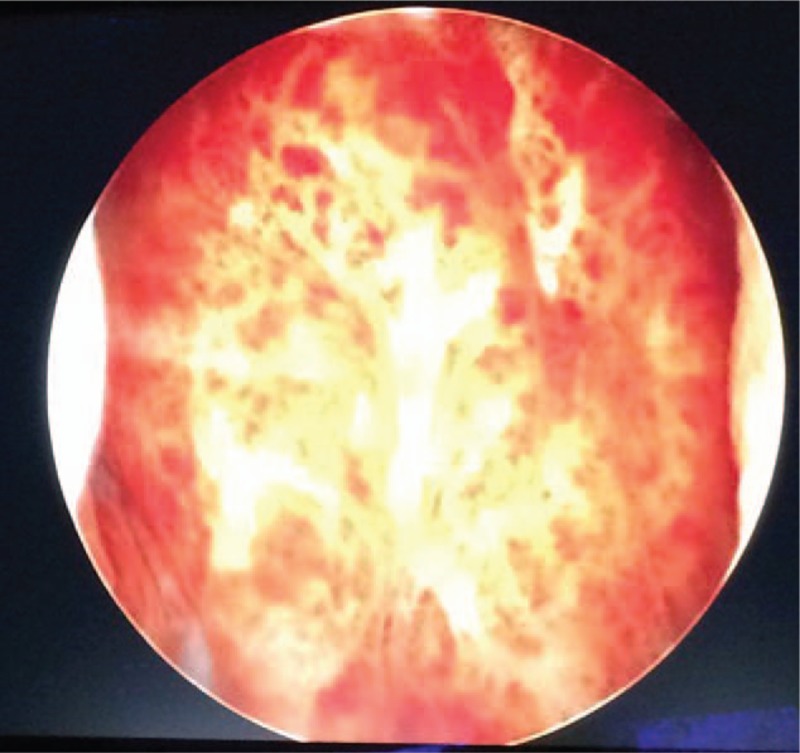
The bladder wall infiltrated by squamous cell carcinoma (cystoscopic view).

**Figure 5:**
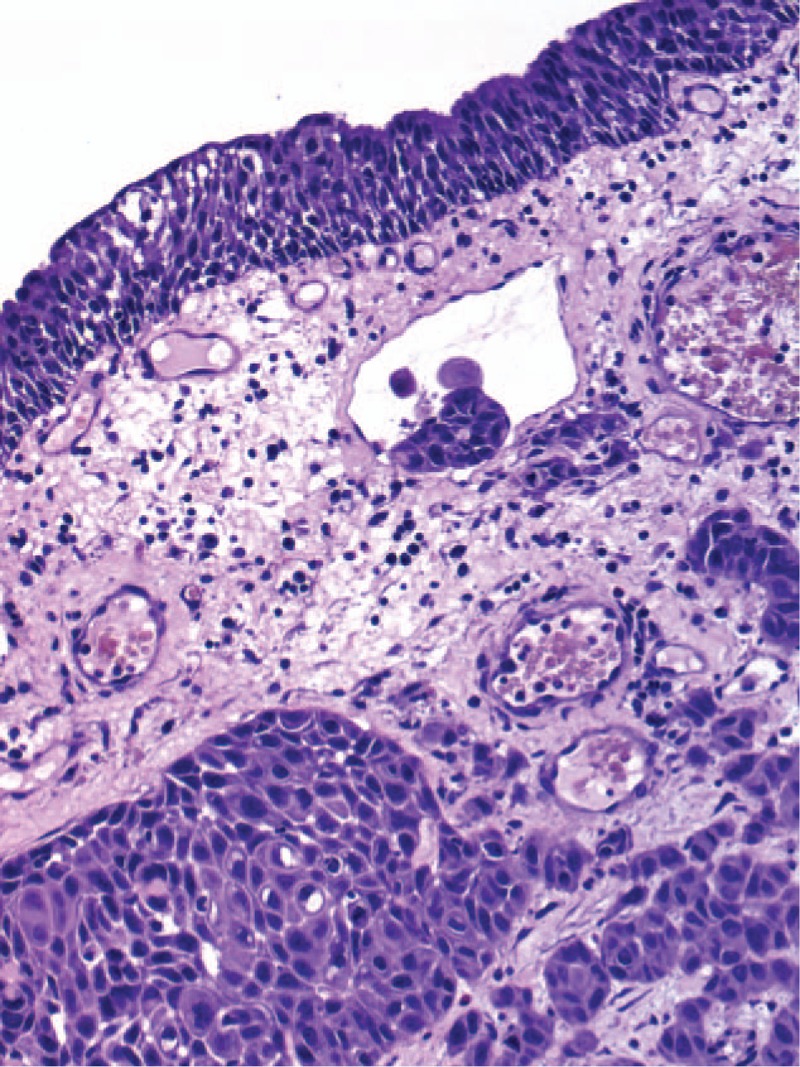
H&E, objective ×10. This diagnostic bladder biopsy shows unremarkable urothelial epithelium (top) with underlying, within submucosa, well-differentiated squamous cell carcinoma (buttom). Well-defined vascular invasion is seen.

Cystoscopy-mucosal lesions were found in the bladder (Fig. 4). The diagnostic biopsies were collected from the urethra and the bladder (Fig. 5).

Symptomatic treatment included: InsulinueGensulin R t.i.d. (2–6 units)sc, Amlodypinum 2 × 10 mg, 0.9%NaCl 2 × 500 ml iv., Clexan (Enoxaparinumnatricum) 2 × 0.6 sc, Ramiprilum 5 mg 1 × 1, Captoprilum 25 mg 1 × 1.

The patient was disqualified from surgery due to the severity of the disease, lymph node metastases, and coexisting comorbidities. Only palliative nephrostomy was performed.

The patient signed informed consents. In our case the patient accepted regular and proved diagnosis and therapy in Clinical Department of Urology, so the ethical approval was not necessary.

## 3 Discussion

The paper presents the vaginal cancer coexisting with ICS-IV prolapse in a 69-year-old woman. Other papers described vaginal carcinoma with coexisting prolapse stage III in ICS in 15 cases, while stage IV only in 3 cases. The age of the patients in 19 described cases was 44 to 83 years. The data from the literature is presented in Table 1.^[1,3–14,15]^ In the available papers, only 3 publications reviewed the literature: both Ghosh and Wang analyze data from 5 papers,^[10,13]^ while Kowalski analyzes 8 papers.^[14]^ In the presented case bilateral hydronephrosis occurred. According to the literature, hydronephrosis may be due to profound static disorders.^[16–19]^ In 5 papers (6 cases) of vaginal cancer and prolapse, hydronephrosis was also reported.^[1,3–5,15]^ In our case, bladder involvement was diagnosed on the basis of the diagnostic biopsies collected in the cystoscopy. Bladder involvement in vaginal cancer qualifies the case as stage IVa FIGO.^[20]^ In the studies on vaginal carcinoma without prolapse, the incidence of stage IVa is only estimated at 8.98%.^[20]^ In our case, as in Sonkusare's case,^[6]^ kidney failure has occurred. According to the author, this failure was due to prolonged prolapse and inferior obstruction.^[6]^ In our case, the diabetologist suspected that renal failure was associated with diabetes, in the case described by Sonkusare diabetes was present, as in the present case, for more than 20 years.^[6]^

**Table 1:**
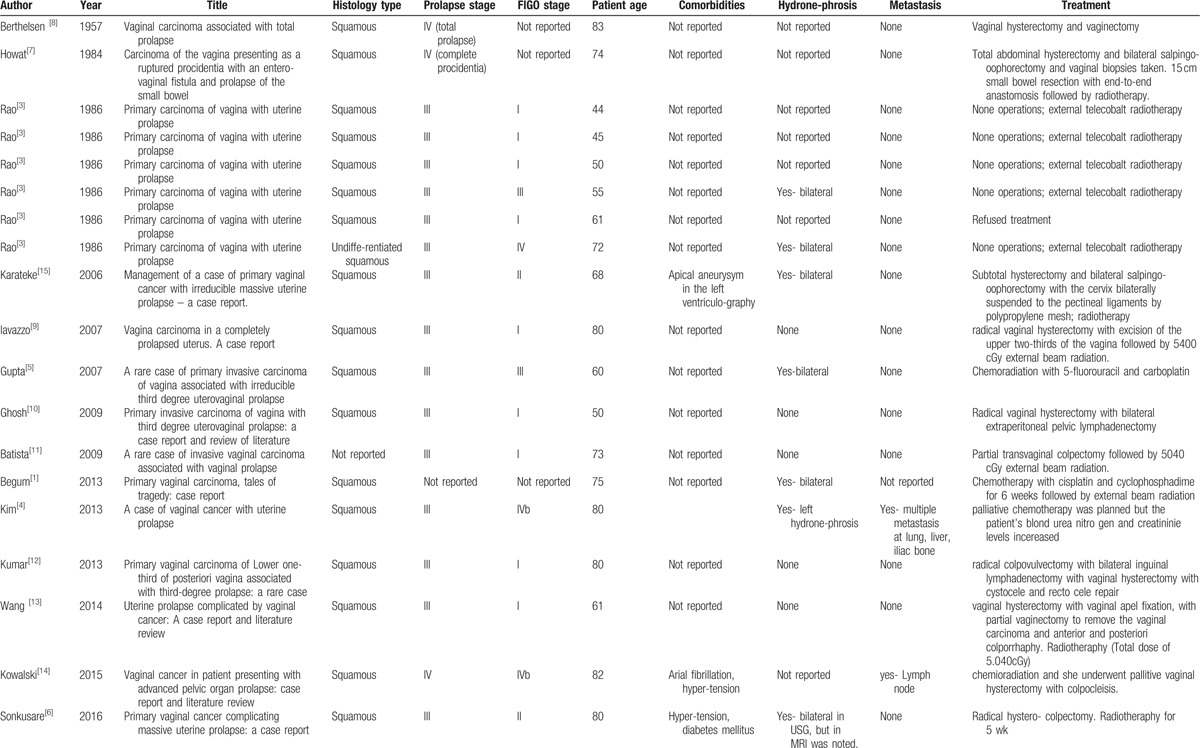
Review of the literature on vaginal cancer with pelvic organ prolapse.

In the presented case, squamous cell carcinoma was found in both vaginal and bladder wall specimens. Interestingly, the cases of reversed metastases from urogenital bladder cancer to the vagina have been reported in the literature.^[21–26]^

Considering the cause of hydronephrosis, the lower obstruction may be due to bladder involvement. Obstructions of this type are found in cervical carcinoma, where hydronephrosis qualifies cervical cancer cases to stage III FIGO.^[27]^ Perhaps in our case this hydronephrosis may be caused by parallel bladder infiltration and prolapse. It cannot be ruled out that diabetes was involved in the development of renal failure.

Literature provides a variety of treatment options for vaginal cancer with prolapse including surgical treatment, radiotherapy, and palliative treatment (Table 1). It should be emphasized that radiotherapy alone in such cases is associated with the complications due to the exposure of hernia to radiation. Rao reports 2 cases of vesicovaginal fistula after radiotherapy,^[3]^ while Howat describes the case in which stoma was necessary after radiotherapy.^[7]^ Among the patients treated surgically, hysterectomy with adnexa and extensive vaginal excision was performed (Table 1).

In the present case due to the advanced stage, accompanying diseases, metastases, the usual, surgical treatment was abandoned and only palliative nephrostomy was used. In the literature only two cases were limited to palliative treatment.

## 4 Conclusion

In the case of coincidence of vaginal cancer and prolapse, there is possibility of bladder involvement and cystoscopic evaluation should be considered.

Coexisting hydronephrosis may be a result of prolapsed and/or bladder involvement.
